# Reflective thinking predicts disbelief in God across 19 countries

**DOI:** 10.3758/s13423-025-02691-9

**Published:** 2025-04-24

**Authors:** Omid Ghasemi, Onurcan Yilmaz, Ozan Isler, Jenny Terry, Robert M. Ross

**Affiliations:** 1 Institute for Climate Risk and Response, University of New South Wales, Sydney, Australia; 2 Department of Psychology, Kadir Has University, Istanbul, Türkiye; 3 School of Economics, The University of Queensland, Brisbane, Australia; 4 School of Psychology, University of Sussex, Brighton, England; 5 Department of Philosophy, Macquarie University, Sydney, Australia

**Keywords:** Belief in God, Cognitive Reflection Test, Intuition, Prime, Reflection

## Abstract

In the present study, we tested three hypotheses about relationships between reflective thinking, intuitive thinking (both measured using the Cognitive Reflection Test; CRT), and belief in God or gods (BiG) in university students across 19 culturally and geographically diverse countries (*n* = 7,771). In support of our first hypothesis, we found a negative relationship between reflective thinking and BiG; and in support of our second hypothesis, we found a positive relationship between intuitive thinking and BiG. Contrary to our third hypothesis, we found no evidence that measuring CRT prior to measuring BiG decreased BiG. Given that this is the first large cross-cultural test of these hypotheses to have a preregistered analysis plan, the first to hold education constant across countries, and the first to use both Bayesian and frequentist methods, these results considerably bolster the evidence in support of the first two hypotheses and against the third hypothesis.

**Supplementary Information:**

The online version contains supplementary material available at 10.3758/s13423-025-02691-9.

## Introduction

In recent decades, there has been growing interest in using cognitive and evolutionary sciences to explain religion, including belief in God or gods (BiG) and lack thereof (Fitouchi et al., 2025; Gervais, 2024; Jackson et al., 2021). A key focus of this literature has been to elucidate relationships between cognitive processes and BiG in terms of an influential dual-process model of reasoning (Baron, 2020; Yilmaz, 2021), which posits two types of cognitive processing (Pennycook, 2023; Saribay & Yilmaz, 2025): Type 1, which tends to be intuitive, automatic, and low-effort, and Type 2, which is more reflective, controlled, and high-effort. It has been proposed that religion is rooted in intuitive assumptions, and intuitive thinking tends to reinforce BiG, while reflective thinking tends to inhibit it (Norenzayan & Gervais, 2013).

### Cognitive reflection and BiG

The classic studies testing the intuitive religious belief hypothesis were published by three independent research groups in 2012 (Gervais & Norenzayan, 2012; Pennycook et al., 2012; Shenhav et al., 2012). All three groups used the same measure of intuitive versus reflective thinking, the Cognitive Reflection Test (CRT; Frederick, 2005), and found the predicted negative association between reflective thinking and BiG, which is consistent with the theory that a disposition to engage in reflective thinking makes people more likely to question religious claims (Baron, 2020; Yilmaz, 2021). While these initial studies were conducted with participants from Canada and the USA, which are two highly WEIRD (Western, Educated, Industrialized, Rich, Democratic) societies (Henrich et al., 2010), subsequent research on participants from more diverse societies has often, but not always, found converging results (Baron, 2020; Pennycook et al., 2016; Yilmaz, 2021).

Only three large cross-cultural studies have been published that examine this hypothesis.^1^ The first, conducted by Gervais et al. (2018), examined 13 countries (*n* = 3,461). Overall, this study revealed a small negative association between reflective CRT score and BiG, but this association was not reliably observed across all countries. In particular, evidence for the predicted negative association was not found in the UK, Czechia, the Netherlands, or New Zealand, and there was evidence for a positive association in the UK.

The second of these studies, conducted by Stagnaro and Pennycook (2025), recruited participants from 16 countries (*n* = 20,216). Overall, they found a small negative association between CRT scores and BiG. However, like Gervais et al. (2018), they found considerable heterogeneity in effect sizes with three countries not exhibiting evidence for an association: Nigeria, Egypt, and Saudi Arabia. Notably, the countries that failed to support the intuitive belief hypothesis were particularly religious, which is in tension with the findings of Gervais et al. (2018) which found that it was highly secular countries that failed to support the hypothesis.

The third of these studies, conducted by Byrd et al. (2025), included participants from eight countries (*n* = 65,873) and differed from the other two large cross-cultural studies by measuring general religiosity instead of BiG. Nonetheless, overall, this study found a small negative association between reflective CRT scores and religiosity. While the reported analyses did not formally test which particular countries provided support for the hypothesis, examining a plot reported in the paper reveals that associations between reflective CRT scores and religiosity were directionally negative in seven countries (with slopes appearing to vary considerably). Interestingly, the one country where the association was directionally positive was India, which contrasts with the results of Gervais et al. (2018) and Stagnaro and Pennycook (2025) which both found evidence for the predicted negative relationship in this country.

### Outstanding questions

Five concerns about the robustness of relationships between BiG and cognitive reflection warrant careful attention. First, there are two approaches to scoring the CRT: a reflective scoring, which is a sum score of correct CRT responses, and an intuitive scoring, which is a sum score of intuitive yet incorrect CRT responses. The three large cross-cultural studies, like most studies in this literature, only examined reflective CRT scoring, but an intuitive scoring, which has occasionally been used in smaller studies (e.g., Shenhav et al., 2012), may exhibit stronger and more robust associations with BiG.

Second, in a single-country experimental study, Finley et al. (2015) reported that administering the CRT prior to belief elicitation can prime reflective thinking and, thus, temporarily supress BiG. This finding provides rare direct evidence that manipulating reflective thinking has a causal impact on BiG. However, this result has failed to replicate (Bahçekapili & Yilmaz, 2017; Pennycook et al., 2016). Nonetheless, if priming effects are subtle, effect sizes could be very small and inconsistent, meaning that large cross-cultural studies might be needed to reliably identify evidence of an impact of priming. Unfortunately, none of the three large cross-cultural studies discussed above randomised the order of presentation of the CRT and the BiG question, so they cannot be used to test this priming hypothesis.

Third, studies in this literature have not used comparable participant samples across different countries. Gervais et al. (2018) used a mixture of university student and general community samples; the samples in Stagnaro and Pennycook (2025) were collected using survey participant providers that have been shown to have data quality issues (Stagnaro et al., 2024); and Byrd et al. (2025) reanalysed data collected from a variety of related studies conducted over 9 years. Crucially, none of these studies recruited samples across countries with comparable or systematically matched socioeconomic characteristics, such as educational background, and there is compelling evidence that lack of consistency in terms of sampling across countries can seriously curtails the appropriateness of cross-cultural comparisons (Ghai et al., 2024; Schimmelpfennig et al., 2024; White & Muthukrishna, 2024). This lack of consistency and control in sample selection might have contributed to the conflicting results among these three earlier cross-cultural studies with respect to which countries provided support for the intuitive religious belief hypothesis and which did not.

Fourth, the cross-cultural study by Gervais et al. (2018) used Bayesian methods whereas the cross-cultural studies by Stagnaro and Pennycook (2025) and Byrd et al. (2025) used frequentist methods. Given that Bayesian and frequentist methods make different assumptions and focus on different statistics when making inferences, it is uncertain whether alternative methodological approaches might have contributed to varying effect size estimates and conflicting results across studies. Indeed, conflicting inferences between Bayesian and frequentist methods are not uncommon (Flores et al., 2022; Nalborczyk et al., 2019).

Fifth, none of the three observational cross-cultural studies (Byrd et al., 2025; Gervais et al., 2018; Stagnaro & Pennycook, 2025) nor the single-country experimental priming study (Finley et al., 2015) were preregistered. Given concerns about the credibility and replicability of research in the cognitive and evolutionary sciences of religion (Gervais, 2024; Hoogeveen & van Elk, 2020; McPhetres et al., 2021; Ross & McKay, 2021; van Elk et al., 2015; Watanabe & Laurent, 2020), it is important that the robustness of relationships between reflective thinking, intuitive thinking, and BiG be examined in a large, preregistered, cross-cultural study.

### The present study

We examined data from 19 countries (*n* = 7,771) to interrogate the cross-cultural robustness and consistency of the relationships between CRT and BiG scores, while addressing the five aforementioned limitations of the existing literature. First, we measured performance on the CRT using both reflective scoring and intuitive scoring. Second, we randomised the order of presentation of the CRT and BiG to test whether answering the CRT first reduces BiG. Third, we achieved increased comparability of educational backgrounds across countries by recruiting university students. Fourth, we used both Bayesian and frequentist methods to examine the extent to which results are robust to analytical approach. And fifth, we preregistered our hypotheses and analysis plans.

## Method

Our data exclusion procedure, hypotheses, and analytical approach were preregistered. The preregistration, raw data, study materials, and analysis code can be found on the project’s Open Science Framework webpage (https://osf.io/5hrtm/). The present project was preregistered after data collection was finished for the paper (Terry et al., 2023) from which we received our data. Prior to developing the preregistration, we examined a partial version of the raw dataset that retained country and basic demographic variables (age and gender) but, importantly, withheld CRT and BiG data. This enabled us to develop our research questions and statistical models (in particular, to consider the number of participants in each country) without any risk of biasing our analyses by having access to the key variables. We did not perform a power analysis because the dataset was preexisting.

### Participants

For a participant to not be screened out of the survey, they needed to indicate at the very beginning of the survey that 1) they are an undergraduate student and 2) they previously studied or are currently studying statistics as part of the research methods training on their degree course, but statistics was not their major subject. The dataset contains survey responses from a large sample of 12,570 students from 100 universities across 35 countries and 21 languages. Data collection took place between January 2021 and September 2021.

Follow-up questions revealed that most participants were majoring in a social science, with psychology being the most common degree major (69.7%), followed by business and finance (6.1%), and education (3.2%). A much smaller percentage of participants were majoring in STEM subjects, with the greatest numbers being in health and medical sciences (2.2%) and computer science (1.0%). Contrary to participant reports in the screener questions, 0.02% of participants indicated in a follow up question that they were postgraduate students (we did not remove these participants because they comprise a very small percentage of the data and to avoid deviating from our preregistered analysis plan).

After implementing our exclusion criteria, as detailed below, we arrived at a total of 7,771 participants from 19 countries and 61 universities using 12 different languages (6,196 women; 1,451 men; 74 nonbinary, another gender, or preferred not to answer; and 50 did not answer); 5,803 participants had ages ranging from 18 to 21 years, 1,287 participants from 22 to 25 years, 434 participants 26 years or older, and 247 participants did not answer. A world map identifying the countries included in the study, with the corresponding participant counts, can be found in the Supplementary Materials (Figure S1).

### Materials

The survey included a variety of questions focused on mathematics and statistics attitudes and had a median completion time of 30 min. The CRT and the BiG measures appeared near the end of the survey (for the full survey see Terry et al., 2023). The study was conducted using the Qualtrics survey software, which was used for randomising the order of survey questions including the order of presentation of the three CRT items and whether the CRT was presented first or BiG question was presented first.

Cognitive reflection was assessed using the CRT, which comprises three “trick questions” that each cue an incorrect intuitive response that can be overridden to identify the correct answer by engaging in reflective deliberation (Frederick, 2005; Meyer et al., 2024). For example, “A bat and a ball cost $1.10 in total. The bat costs $1.00 more than the ball. How much does the ball cost?” For most people, an intuitive answer that comes readily to mind is 10 cents. However, further deliberation reveals that the intuitive response is incorrect and that the correct answer is 5 cents. The CRT can be scored in two ways: a reflective CRT scoring (the sum of correct CRT responses, ranging from 0 to 3) and an intuitive CRT scoring (the sum of intuitive incorrect CRT responses, also ranging from 0 to 3). We used a version of the CRT developed by Shenhav et al. (2012) that comprises of three new items that are very similar to the original items because items from the original version, particularly the bat and ball problem, have become well-known.

BiG was measured using a single question from Shenhav et al. (2012) which asked, “How strongly do you believe in God (or gods)?” Participants were instructed to provide a numerical response ranging from 0 (indicating absolute certainty that God or gods do not exist) to 100 (indicating absolute certainty that God or gods do exist).

We employed a preregistered multistep approach to exclude participants who did not complete the full survey, or did not answer key questions, or provided low-quality data. First, we excluded those participants who did not receive the CRT or BiG questions, either because they were not presented with the BiG question (*n* = 100 participants from Saudi Arabia) or because they had quit the experiment before encountering these questions (*n* = 1,497 participants across all countries). Next, those participants who did not respond to the final question of the survey, which indicates that they had not reached the end of the survey (*n* = 800). Next, those with BiG responses outside the range of 0 to 100 (*n* = 33). Next, those participants who failed three or more of six attention checks (*n* = 1,291).^2^ Next, all participants from Türkiye (*n* = 834) due to the accidental removal of attention check questions during survey translation into Turkish. Next, those participants who selected “No, I have not answered the questions carefully and truthfully” to an amnesty question on the final screen of the survey which asked, “Please indicate whether you feel you have answered the previous questions carefully and truthfully” (*n* = 92). Next, those participants from countries with sample sizes below 100 (*k* = 16, *n* = 796) to avoid country-level estimates of statistical associations that are very imprecise. Finally, all participants from the University of La Laguna due to unexplained anomalies in the CRT data at this site (*n* = 190).^3^ Overall, after applying all our exclusion criteria, 7,771 participants from 61 universities across 19 countries were retained for analysis.^4^

### Analysis

We tested three preregistered hypotheses using both Bayesian and frequentist approaches:

- Hypothesis 1. Reflective (i.e., correct) CRT score predicts lower BiG score.
- Hypothesis 2. Intuitive CRT score predicts higher BiG score.
- Hypothesis 3. BiG score is lower when the CRT is presented before the BiG measure compared with when the CRT is presented after the BiG measure.

For the Bayesian analyses, we developed three distinct hierarchical regression models with BiG as the dependent variable. The models varied by including one of the following predictors: reflective CRT score (testing Hypothesis 1), intuitive CRT score (testing Hypothesis 2), or order of the CRT measure and the BiG measure (testing Hypothesis 3). For testing hypotheses, we estimate 95% highest density intervals (HDIs) overall and for each country.

All three models included a random intercept for country and a random slope for the respective CRT score predictor, allowing for variability in the relationship between CRT scores and BiG across countries. The prior for the intercept parameter was set to follow a normal distribution with the mean and standard deviation of the observed data. For the fixed-effect coefficients, normal priors were centred at zero with a standard deviation of 5 for the first two models and a standard deviation of 10 for the third model. The standard deviation of the random effects and residual standard deviation followed Cauchy distributions with scale parameters of 3 and 2, respectively. Additionally, the prior for the correlation matrix was modelled using an LKJ distribution with a shape parameter of 2. Markov chain Monte Carlo (MCMC) settings were consistent across all models, including four chains each with 30,000 iterations after 3,000 warmup draws.

For the frequentist analyses, we carried out three random-effect meta-analyses to test each of the three hypotheses. The first model examined the correlation between reflective CRT score and BiG (Hypothesis 1); the second model examined the correlation between intuitive CRT score and BiG (Hypothesis 2); and the third model estimated the standarised mean difference (Hedges’s g) between measuring the CRT or BiG first (Hypothesis 3). All frequentist analyses used two-tailed tests. In all cases, we used the restricted maximum likelihood method to estimate the value of tau squared (τ^2^) and applied the Knapp–Hartung adjustment to accommodate uncertainty in our estimation of between-study heterogeneity. For testing hypotheses, we estimate 95% confidence intervals (CIs) overall and for each country.

While we did not preregister an intention to examine the internal consistency of measures, we report Cronbach’s alpha and omega total. When considering internal consistency, we focus on omega total because Cronbach’s alpha is known to make assumptions that might not be realistic (McNeish, 2018).

All analyses were conducted using R (R Core Team, 2024); with *tidyverse* for data cleaning and visualization (Wickham et al., 2019); *brms* (Bürkner, 2017), *marginaleffects* (Arel-Bundock, 2024), and *tidybayes* (Kay, 2023) for Bayesian hierarchical analysis; and *meta* (Balduzzi et al., 2019) for frequentist meta-analysis.

## Results

Consistent with earlier work (e.g., Bialek & Pennycook, 2018; Primi et al., 2016), CRT had relatively poor internal consistency for most countries (see Table S1).^5^ Nonetheless, we follow our preregistered analysis plan (and standard practices in the literature) and use CRT sum scores for testing our hypotheses, for three main reasons. First, the internal consistency of the CRT tends to improve considerably when the number of items is increased from three to six (e.g., Newton et al., 2024; Primi et al., 2016), which suggests that the poor internal consistency of the three-item CRT is due to it being an extremely short measure, rather than problems with the items themselves. Second, each CRT item has an objectively correct and mathematically simple answer (Frederick, 2005), meaning that there is limited scope for CRT items to be measuring different underlying psychological constructs. Third, a vast literature has found that low reflective CRT sum scores predict a remarkable variety of reasoning errors and epistemically suspect beliefs (Pennycook, 2023; Saribay & Yilmaz, 2025).

### Bayesian analyses

**Fig. 1:**
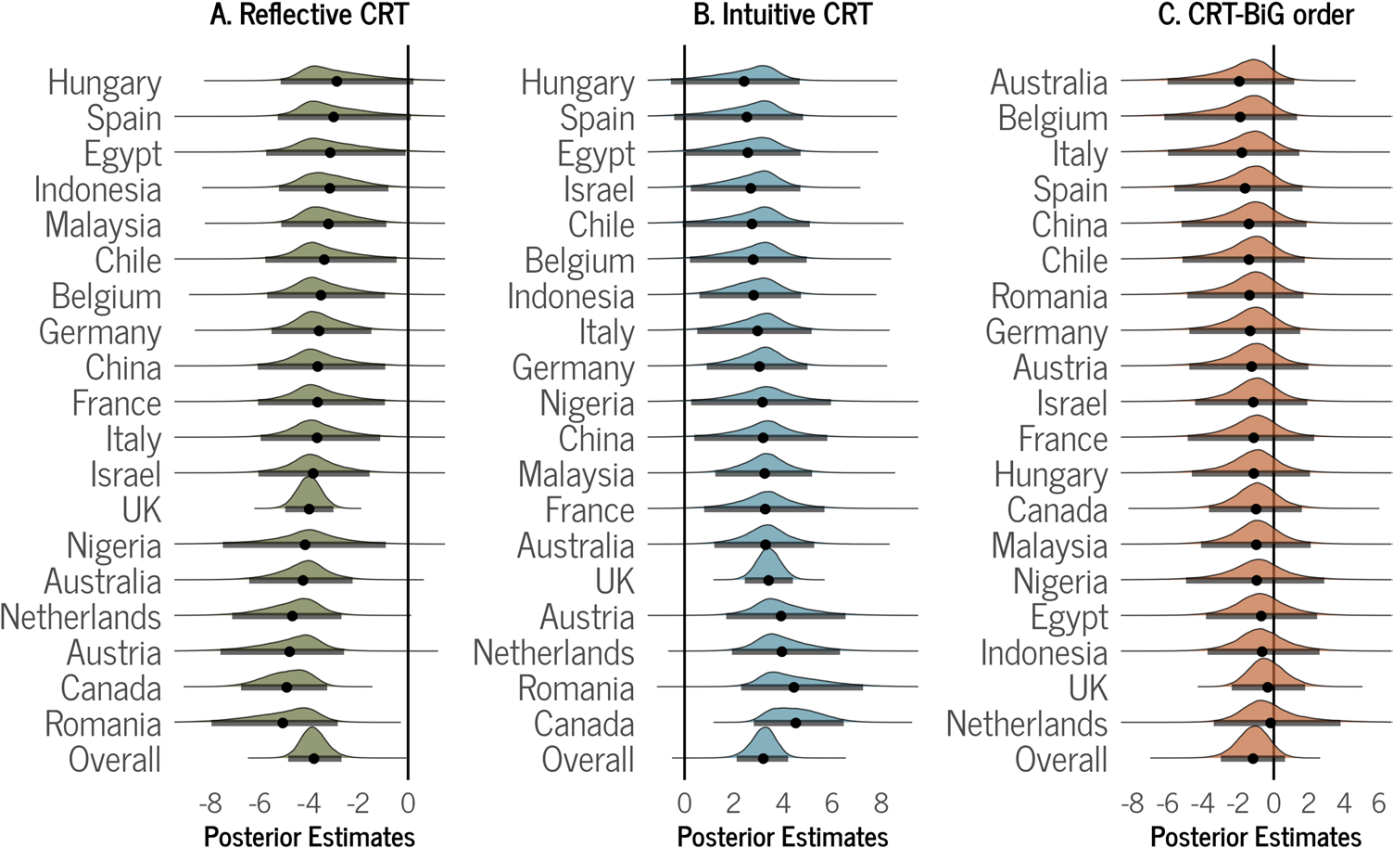
Overall and country-level Bayesian posterior estimates (beta) of predictors. Panel **A** shows the posterior distributions of the average marginal means of reflective CRT score in predicting BiG (Hypothesis 1). Panel **B** shows the posterior distributions of the average marginal means of intuitive CRT score in predicting BiG (Hypothesis 2). Panel **C** shows the average marginal mean for the differences between the reflection group and control group (Hypothesis 3). The vertical lines represent the null value of zero, and the error bars within the distributions represent the 95% highest density intervals (HDIs)

The Bayesian analyses revealed that reflective CRT score has a negative predictive relationship with BiG, β = − 3.83, 95% HDI [− 4.88, − 2.71]. Notably, more than 99.9% of the posterior draws fall below the null value of zero. As shown in Fig. 1A, aside from Hungary and Spain, the posterior estimates are credibly negative, with their 95% HDIs excluding zero as a credible value. In Bayesian models the width of the HDIs for each country is influenced by the number of participants. Consequently, countries with larger participant numbers, such as the UK and Canada, tend to exhibit narrower HDIs, indicating more precise estimates (see Table S2 for a detailed summary of posterior estimates of all models and Fig. S10 for trace plots of main effects for Bayesian models in supplementary materials).

Additionally, our Bayesian analyses revealed that intuitive CRT score is a positive predictor of BiG, β = 3.20, 95% HDI [2.12, 4.21]. Again, more than 99.9% of the posterior draws exclude zero. In most countries, the estimates are credibly positive. However, in certain countries, where the sample size is relatively small, the wide 95% HDIs either overlap with zero (Hungary, Spain, and Chile) or only marginally exclude it (Egypt, Israel, and Belgium).

The results also indicate that there is no credible difference in BiG ratings depending on whether it was measured after (mean = 50.4, 95% HDI [39.2, 61.8]) or before the CRT measure (mean = 51.6, 95% HDI [40.5, 62.8]), β = − 1.18, 95% HDI [− 3.01, 0.64]. As shown in Fig. 1C, the 95% HDIs for the overall distribution encompass zero as a credible value, with 9.3% of the posterior draws falling above zero. Similarly, none of the estimates for countries provided credible evidence of a difference, as they all comfortably included zero as a credible value.

In summary, the Bayesian analyses supported a negative relationship between reflective CRT score and BiG (Hypothesis 1), as well as a positive relationship between intuitive CRT score and BiG (Hypothesis 2), but there was little evidence that presenting the CRT before BiG question decreases BiG (Hypothesis 3).

### Frequentist analyses

**Fig. 2:**
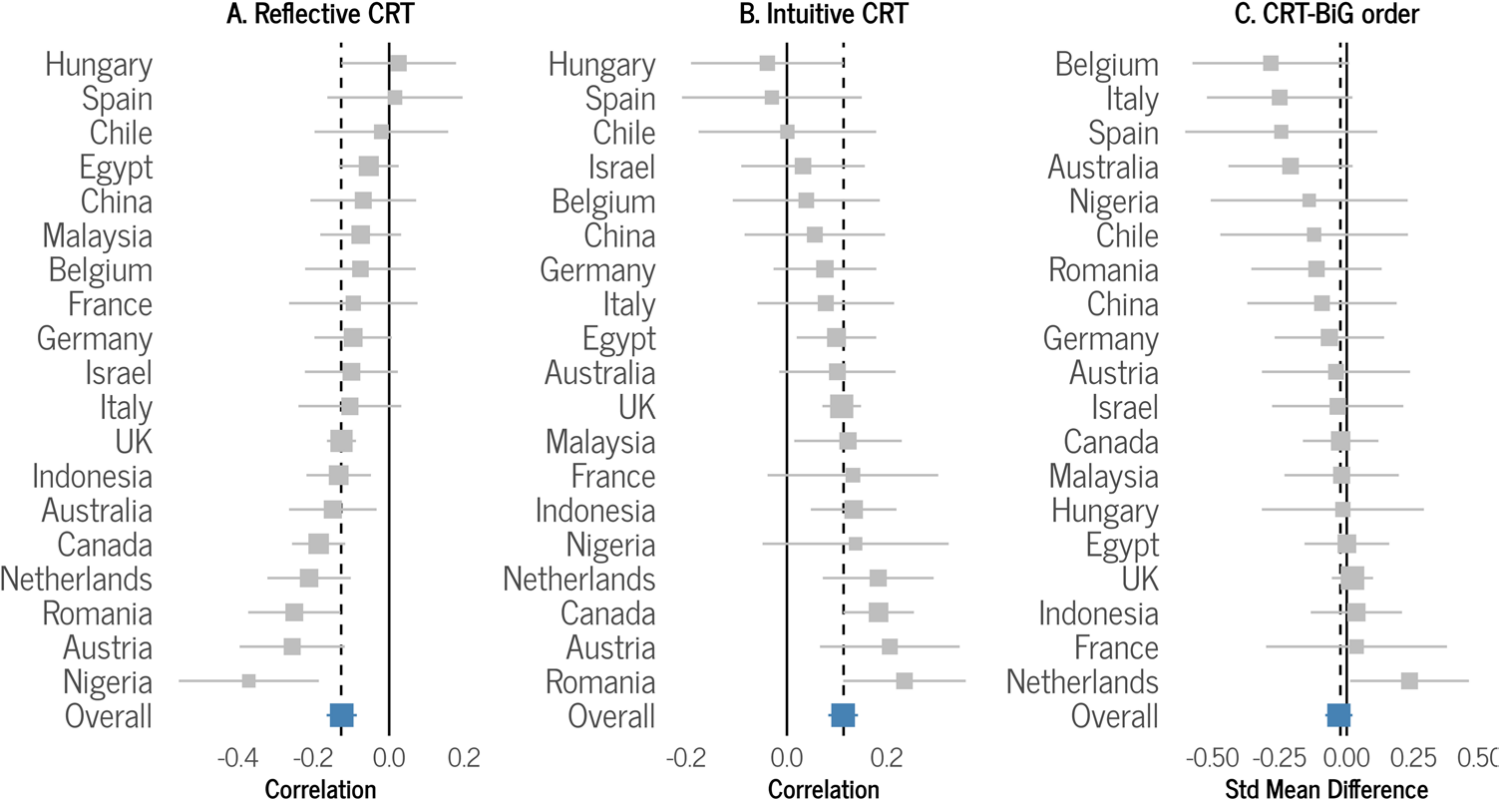
Forest plots illustrating random effect meta-analyses, depicting overall and by-country coefficients (solid squares) with their respective confidence intervals (error bars). Panel **A** shows the results for the correlation between reflective CRT score and BiG (Hypothesis 1). Panel **B** shows the correlation between intuitive CRT score and BiG (Hypothesis 2). Panel **C** shows the standardized mean differences between the measuring CRT first group and the measuring BiG first group (Hypothesis 3). The size of the squares corresponds to the weight of each country in the calculation of the pooled effect size. The dashed vertical line represents the meta-analytic effect size, the solid vertical line represents the null value of zero, and the error bars represent 95% Confidence Intervals (CIs)

The frequentist analyses revealed a negative correlation between reflective CRT score and BiG, pooled *r* = − 0.13, 95% CI [− 0.16, − 0.09], *p* < 0.001. This indicates that individuals with greater cognitive reflection tend to exhibit lower levels of BiG. Our analysis also found statistically significant and moderate heterogeneity in effect sizes among the countries, *I*^2^ = 45.4%, 95% CI [6.5%, 68.1%], *Q*(18) = 32.97, *p* = 0.017. Figure 2A shows that the correlation is negative, and the 95% confidence interval excludes zero in eight countries, but does not exclude zero in 11 countries (for a more detailed plot, see Fig. S7 in the Supplementary Materials).

The results also revealed a positive correlation between intuitive CRT and BiG, pooled *r* = 0.11, 95% CI [0.09, 0.14], *p* < 0.001. This indicates that individuals with higher intuitive CRT scores tend to exhibit greater levels of BiG. Our analyses did not find statistically significant evidence for heterogeneity across countries, *I*^2^ = 22.3%, 95% CI [0.0%, 55.4%], *Q*(18) = 23.16, *p* = 0.18. Figure 2B shows that the correlation is positive, and the 95% CI excludes zero in eight countries, but does not exclude zero in 11 countries (for a more detailed plot, see Fig. S8 in the Supplementary Materials).

The difference in BiG ratings measured before compared to after CRT measurement did not reach statistical significance, Hedges’s *g* = − 0.03, 95% CI [− 0.08, 0.02], *p* = 0.24. Our analysis did not find statistically significant evidence for heterogeneity across countries, *I*^2^ = 2.6%, 95% CI [0.0%, 50.2%], *Q*(18) = 18.48, *p* = 0.42. Figure 2C shows that the confidence interval excluded zero in the Netherlands, but in the opposite direction to what is predicted, and it did not exclude zero in the other 18 countries (for a more detailed plot, see Fig. S9 in the Supplementary Materials).

In summary, the frequentist analyses were consistent with the Bayesian analyses in supporting a negative relationship between reflective CRT score and BiG (Hypothesis 1), as well as a positive relationship between intuitive CRT score and BiG (Hypothesis 2), and again, no evidence was found that presenting the CRT before the BiG question decreases BiG (Hypothesis 3). One noteworthy difference between the results of the two analyses is that due to *shrinkage* (i.e., the phenomenon that in hierarchical models estimates of lower-level parameters are pulled closer together than they would have been had there not been a higher-level distribution; Kruschke, 2014; McElreath, 2020), the hierarchical Bayesian analyses provided country-level beta estimates that are closer to each other than the country-level correlations from the frequentist meta-analyses. This might help explain why, in the case of Hypothesis 1 and Hypothesis 2, there are several countries for which the 95% HDIs from the Bayesian analyses exclude zero (see Fig. 1) while the 95% CIs for frequentist analyses encompass zero (see Fig. 2). Indeed, sample sizes for several countries appear to be too small to precisely estimate parameters given the small effect sizes typical of this literature. Nevertheless, the overall estimates for the Bayesian analyses and the frequentist analyses are highly consistent.

## Discussion

In support of Hypothesis 1, we found that reflective CRT scores were negatively associated with BiG, and in support of Hypothesis 2, we found that intuitive CRT scores were positively associated with BiG. In both cases, effect sizes were small, and not all countries showed evidence for predicted associations when considered separately. By contrast, we did not find support for Hypothesis 3 because BiG scores did not differ when the BiG question was presented after the CRT compared with before the CRT. For all three hypotheses, Bayesian and frequentist analyses yielded highly consistent results.

### Does reflective thinking predict disbelief in God?

Results in support of Hypothesis 1 and Hypothesis 2 are broadly consistent with the intuitive religious belief hypothesis, which predicts a negative association between reflective thinking and BiG and a positive association between intuitive thinking and BiG. While not all countries provided evidence for the hypotheses when examined separately, importantly, no country provided evidence for a positive association between reflective thinking and BiG.

The small overall effect sizes are broadly consistent with earlier cross-cultural studies (Byrd et al., 2025; Gervais et al., 2018; Stagnaro & Pennycook, 2025). Small effect sizes might be seen to call into question the practical significance of these results. However, it has been argued that small effect sizes are common in psychological science and can be consequential (Funder & Ozer 2019; Gotz et al., 2022). Nonetheless, others have argued that the importance of a small effect size needs to be supported by evidence and psychological theory on a case-by-case basis (Anvari et al., 2023; Primbs et al., 2023). There is an ongoing debate about the practical significance of the small effect sizes found in this particular literature (not practically significant: Gervais, 2024; practically significant: Stagnaro & Pennycook, 2025) and we suggest that richer cognitive models that unpack relationships among intuitive thinking, reflective thinking, and BiG (and other individual differences) need be developed to facilitate interpretation of the practical significance (or lack thereof) of the small, yet strongly supported, associations identified in the current study and earlier studies.

### Does priming analytic thinking reduce belief in God?

Despite having a very large sample size, our study provided no evidence in support of Hypothesis 3—that being exposed to the CRT subtly primes reflective thinking and temporarily attenuates BiG. This finding is contrary to the result of Finley et al. (2015) but is consistent with other studies that have likewise failed to find evidence supporting the impact of this analytic prime on BiG (Bahçekapili & Yilmaz, 2017; Pennycook et al., 2016). More broadly, experimental research examining whether priming reflective thinking causes a decrease in BiG has yielded mixed results (Yilmaz, 2021), with two widely cited priming studies from this literature (Gervais & Norenzayan, 2012; Shenhav et al., 2012) failing to replicate in preregistered replication studies (Camerer et al., 2018; Sanchez et al., 2017; Saribay et al., 2020). Thus, in reporting the first large cross-cultural test of whether this reflective priming manipulation impacts on BiG, the current study makes an important contribution to the growing body of evidence showing that it is difficult to induce reflective or intuitive thinking via subtle primes (Isler & Yilmaz, 2023; Leys, 2024).

### Limitations

Two limitations of the present study are worth highlighting. First, all samples in the present study were drawn from university student participant pools. As we have discussed, the closely overlapping educational backgrounds of participants is a key strength of the current study to the extent that it controls for education across countries. This is important because “random sampling is for all intents and purposes dead” (Bailey, 2024, p. 4), and a crucial weakness of earlier cross-cultural studies of relationships between cognitive reflection and BiG is that they used a variety of different samples which, as is often the case in psychology research, raises concerns about making generalisations to the populations of countries and comparisons among countries (Ghai et al., 2024; Schimmelpfennig et al., 2024). Nonetheless, university students are not representative of the broader societies from which they are drawn, particularly in non-WEIRD countries (White & Muthukrishna, 2024). Thus, while our results can be used to make inferences about the psychology of university students across countries with some degree of confidence, our focus on university students creates a constraint on generalizability (Simons et al., 2017) and further work is needed to examine the extent to which relationships between cognitive reflection and BiG hold for other groups from these countries.

Second, the present study only examined the extent to which reflective thinking predicts low levels of BiG, but several other factors have been proposed to contribute to reducing BiG (Norenzayan & Gervais, 2013). As such, it would be inappropriate to use the results of the current study to infer support for the hypothesised causal impact of reflective thinking on BiG, which, to the best of our knowledge, has yet to be rigorously examined in any observational studies using modern tools of causal inference (Bulbulia, 2024; Rohrer, 2018). Indeed, a recent study of predictors of BiG in the USA found that reflective thinking and exposure to displays of religious commitment from parents during childhood interacted to predict BiG (Gervais et al., 2021), which is suggestive of potentially complex causal relationships among reflective thinking, religious upbringing, and BiG.

### Conclusion

Our study of 7,771 university students across 19 geographically and culturally diverse countries found strong evidence that reflective thinking predicts lower levels of belief in God (or gods), and intuitive thinking likewise predicts higher levels.

## Supplementary Information

Below is the link to the electronic supplementary material.

Supplementary file1 (DOCX 3397 KB)

## Data Availability

https://osf.io/5hrtm/

## Footnotes

1 Each of these three cross-cultural studies tested additional hypotheses concerning the relationship between cognitive reflection and religion. However, below, we summarise only those hypotheses of direct relevance to the hypotheses being tested in the current project.

2 Three or more was chosen based on the consideration that by including six attention check questions, each with five options, the probability that an individual who is responding at random answers fewer than three questions incorrectly is less than 2%.

3 As per the preregistered plan, we compared the proportions of reflective, intuitive, and incorrect responses for each country to identify potential translation or coding errors. We found that 69% of responses from the University of La Laguna were recorded as participants supplying “0” as the answer for the first CRT item, which is neither a correct nor an intuitive response. This is a radically different pattern of responses to other universities (see Fig. S3 in Supplementary Materials).

4 The median number of universities per country was two. The UK was an outlier with data being collected from 22 universities (Indonesia had the second greatest number of universities with data being collected from six).

5 See the Supplementary Materials for a table of descriptive statistics (Table S1); and visualisations of country-level BiG and CRT scores (Fig. S2 and Fig. S3, respectively), country-level correlations between reflective CRT scores and BiG and between intuitive CRT scores and BiG (Fig. S4 and Fig. S5, respectively), and country-level distribution plots when BiG was measured before versus after the CRT measure (Fig. S6). In addition, a reviewer expressed an interest in two exploratory analyses, which we have also added to the Supplementary Materials: Table S3 reports the three hypotheses being tested using frequentist hierarchical models (i.e., instead of frequentist meta-analysis as preregistered), and Fig. S11 and Table S4 report the three hypotheses being tested using frequentist hierarchical models with gender and the interaction between gender and CRT as additional predictors.

## Declarations

### Open practices

Our data exclusion procedure, hypotheses, and analytical approach were preregistered, and our preregistration, raw data, study materials, and analysis code can be found on the project’s Open Science Framework webpage (https://osf.io/5hrtm/).
