# Supplementary material for: Reflective thinking predicts disbelief in God across 19 countries
Source: Psychon Bull Rev. 2025 Apr 24;32(5):2220–9. doi: 10.3758/s13423-025-02691-9 (PMC12425841; doi:10.3758/s13423-025-02691-9)
Supplement: Supplementary file 1 — Supplementary file1 (DOCX 3397 KB) [file 13423_2025_2691_MOESM1_ESM.docx]

# Supplementary Materials for Ghasemi, Yilmaz, Isler, Terry, & Ross (2025) Reflective thinking predicts belief in God across 19 countries. *Psychonomic Bulletin & Review*.


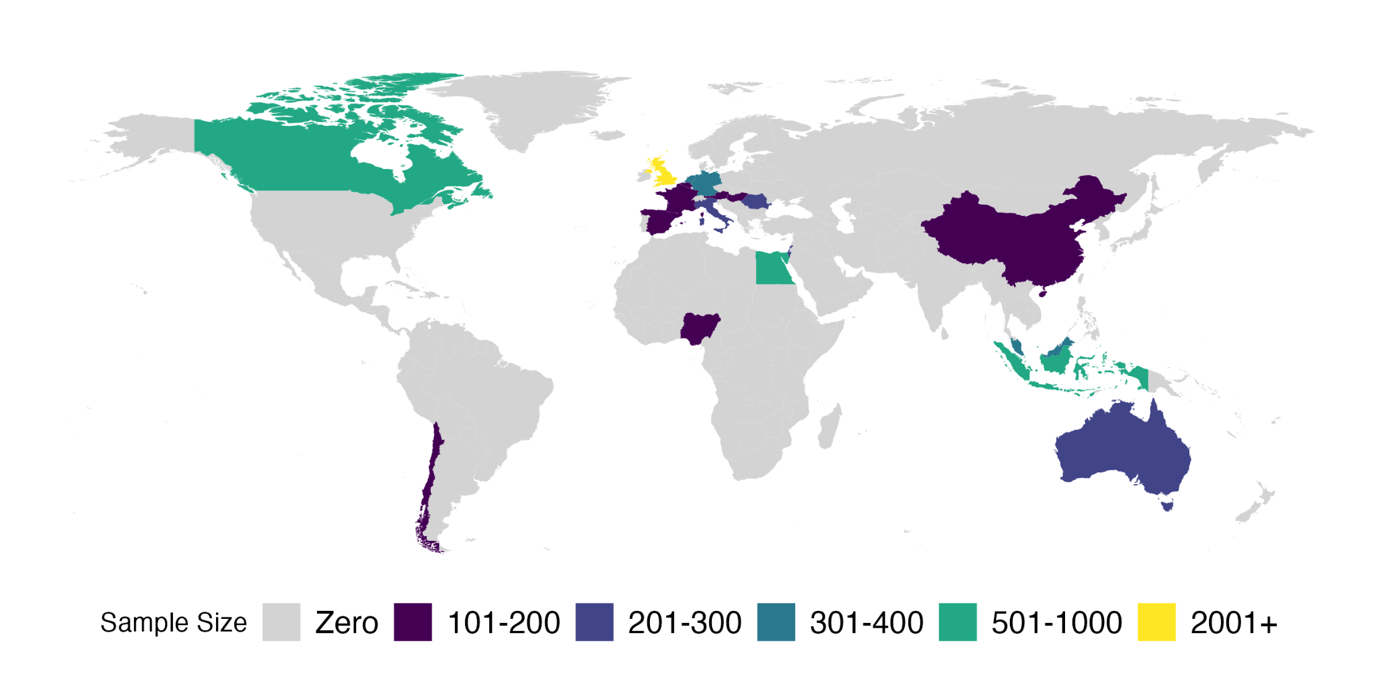


**Figure S1**: A global map depicting the countries included in the study, with the corresponding participant counts.


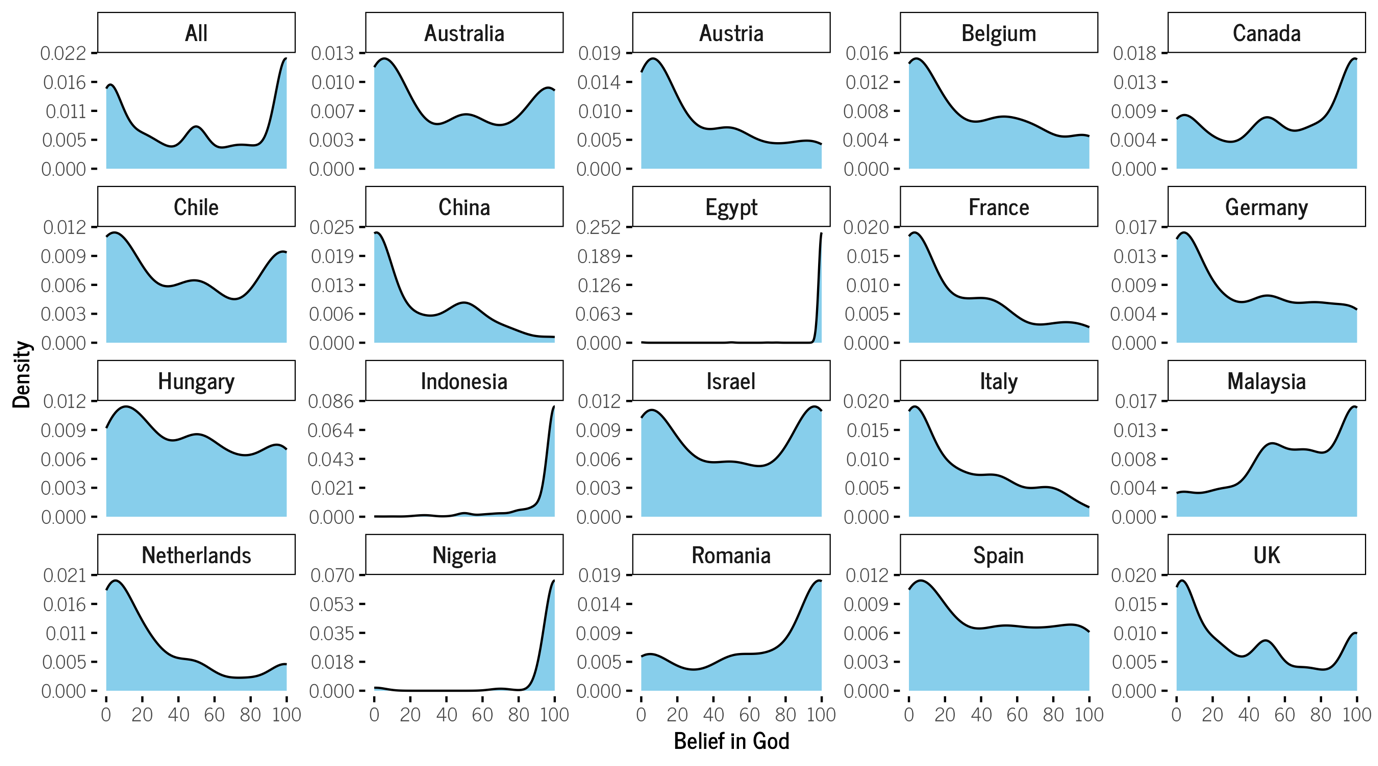


**Figure S2**: Density plots illustrating BiG ratings for the full sample and individual countries. Ratings range from 0 (indicating absolute certainty in the non-existence of God or gods) to 100 (indicating absolute certainty in the existence of God or gods).


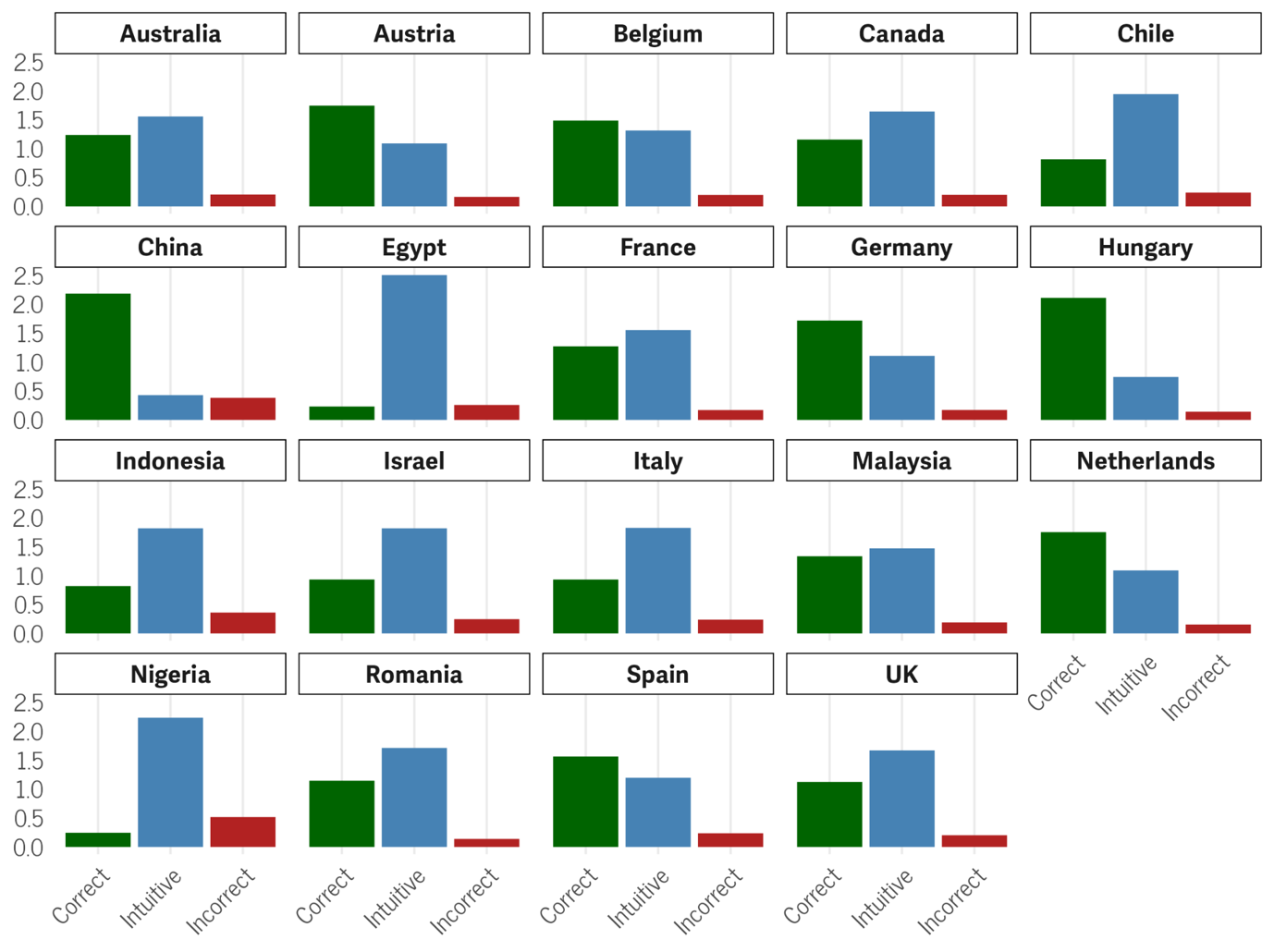


**Figure S3**: Bar plots showing each country’s average number of correct (reflective), intuitive, and non-intuitive incorrect responses to the Cognitive Reflection Test. The test has three items, so the y-axis shows the mean number of responses out of three.


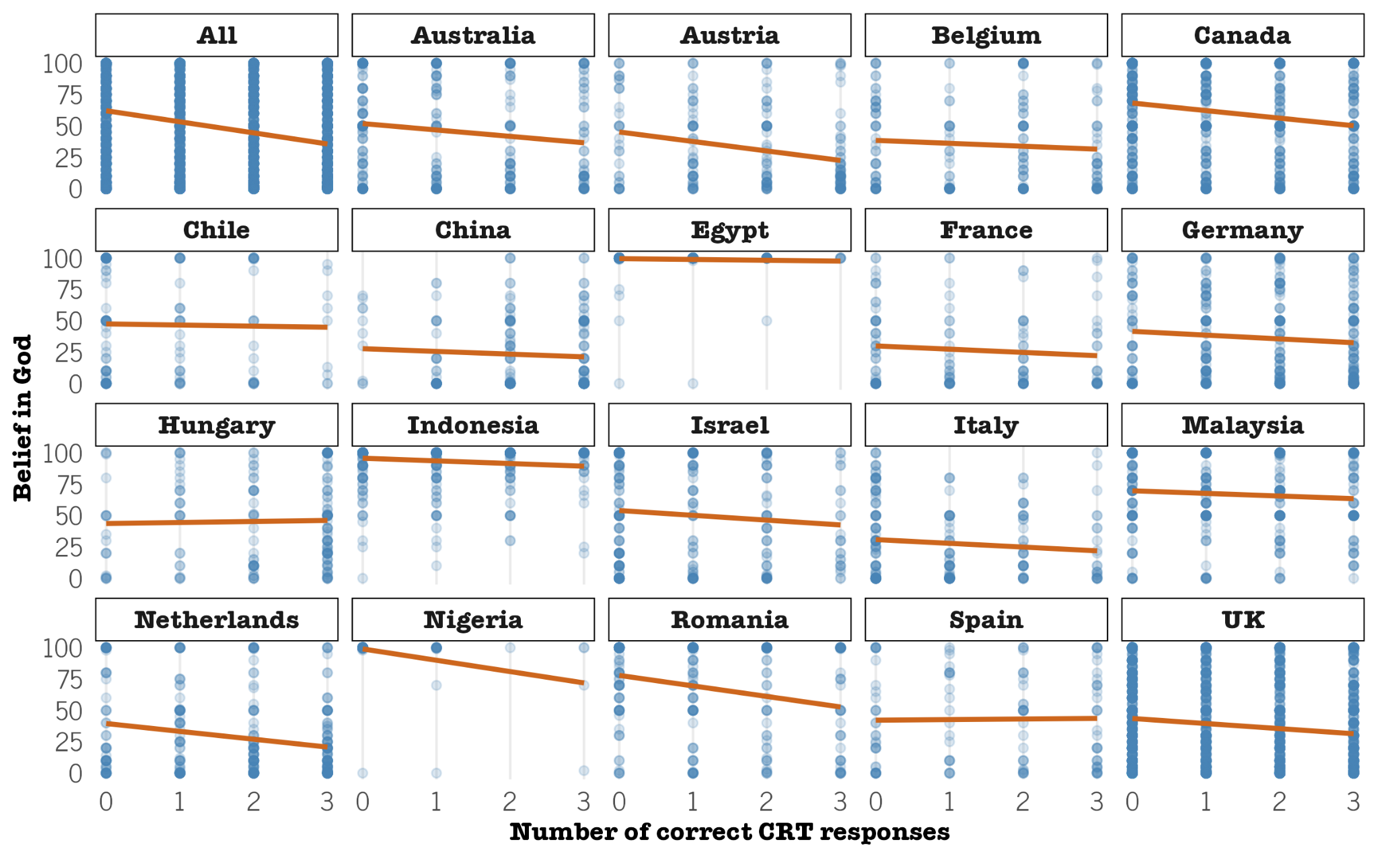


**Figure S4**: Scatterplots with linear regression lines showing the correlation between reflective CRT scores on the x-axis and BiG on the y-axis, both for the overall sample and individual countries.


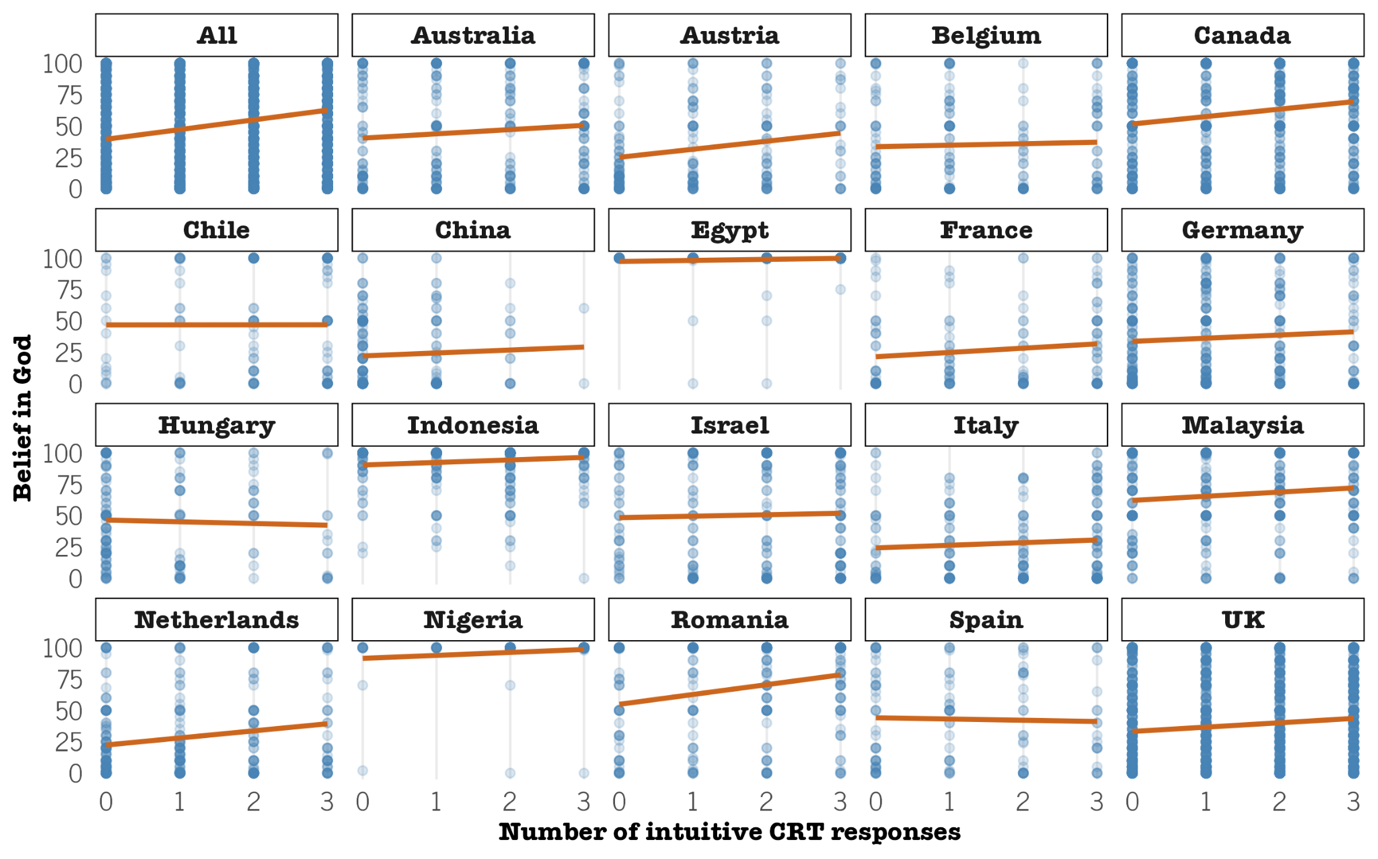


**Figure S5**: Scatterplots with linear regression lines showing the correlation between intuitive CRT scores on the x-axis and BiG on the y-axis, both for the overall sample and individual countries.


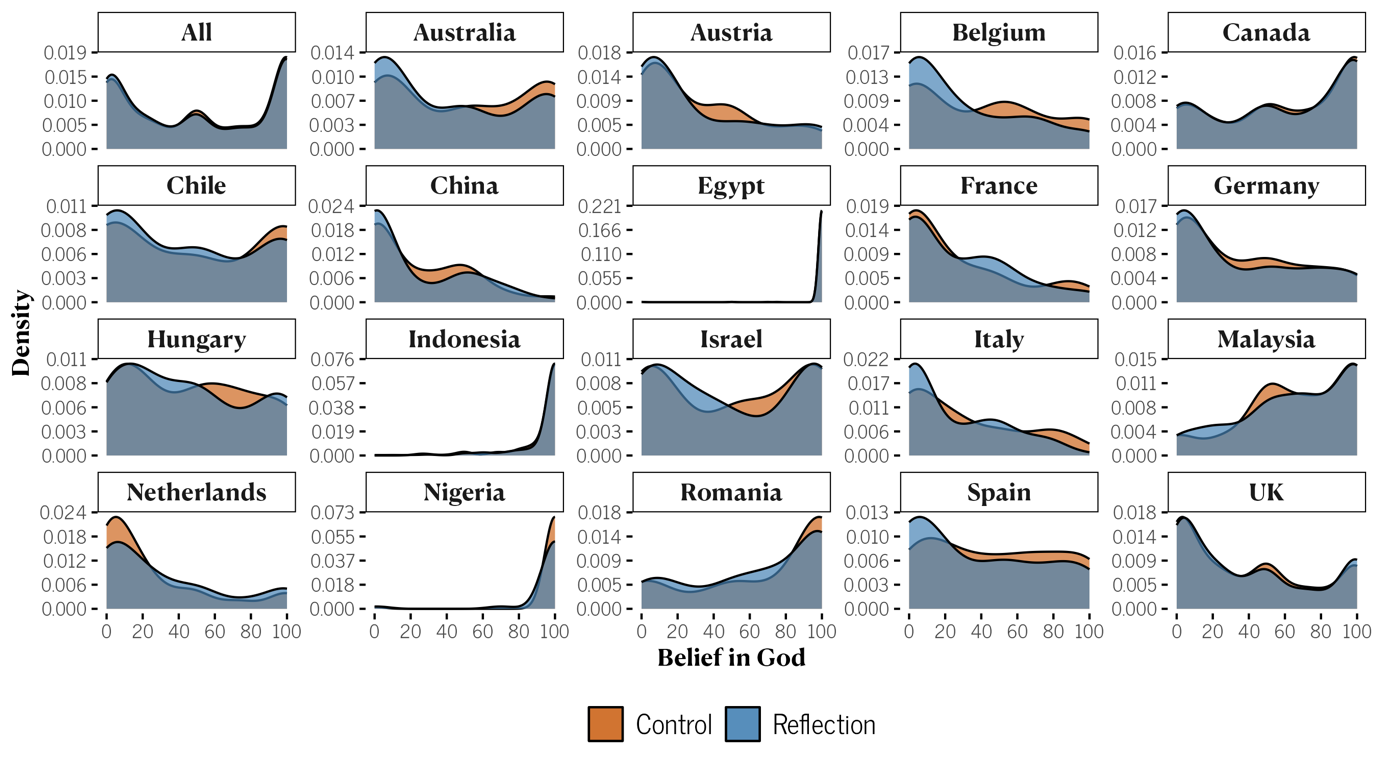


**Figure S6**: Density plots showing BiG ratings for the reflection and control groups, both for the overall sample and individual countries. Ratings range from 0 (indicating absolute certainty in the non-existence of God or gods) to 100 (indicating absolute certainty in the existence of God or gods).


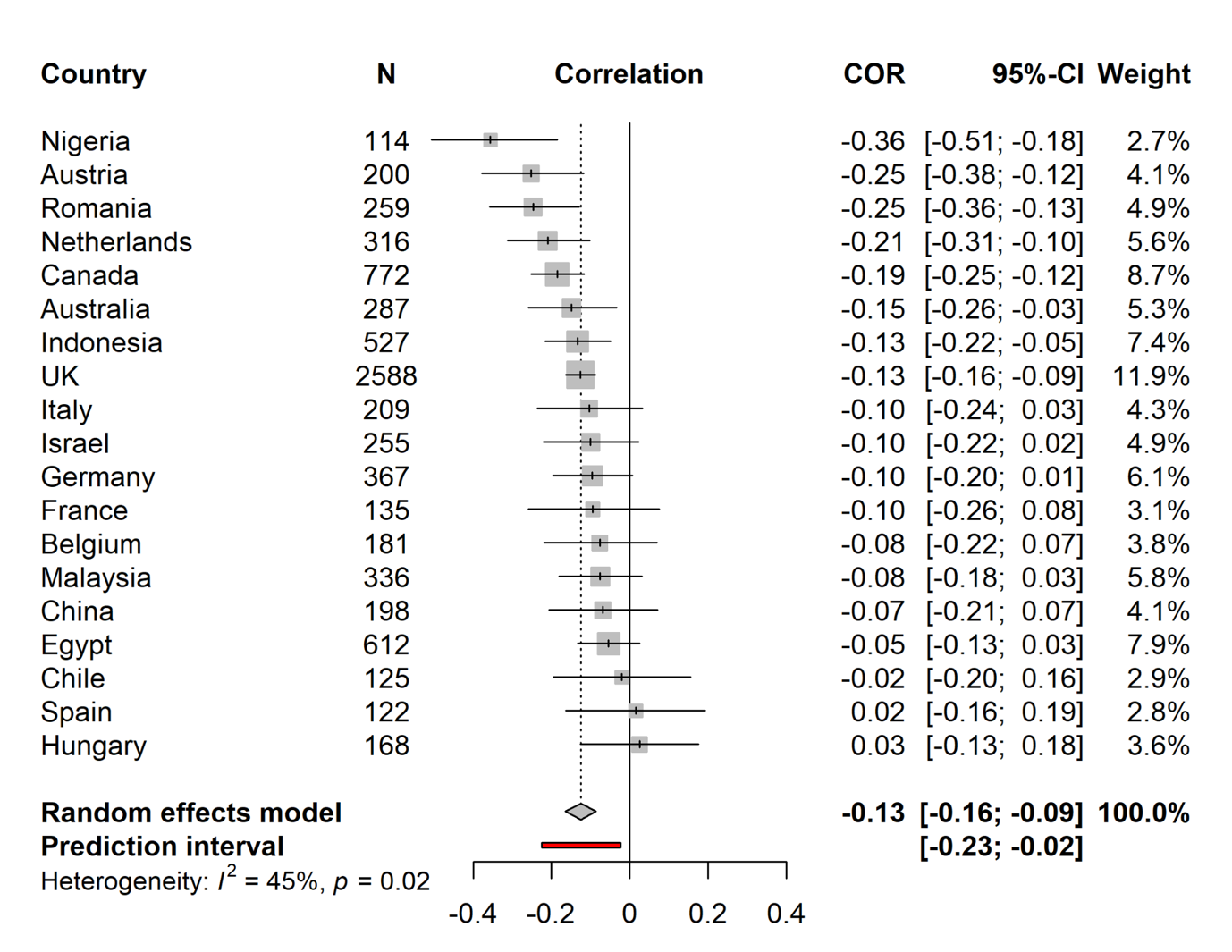


**Figure S7**: Forest plot showing random-effect meta-analysis of the relationship between reflective CRT scores and BiG. This plot presents both overall and country-specific coefficients as solid squares, with their respective 95% Confidence Intervals (CIs) displayed as error bars. The prediction interval reveals that future studies are likely to show a small negative correlation.


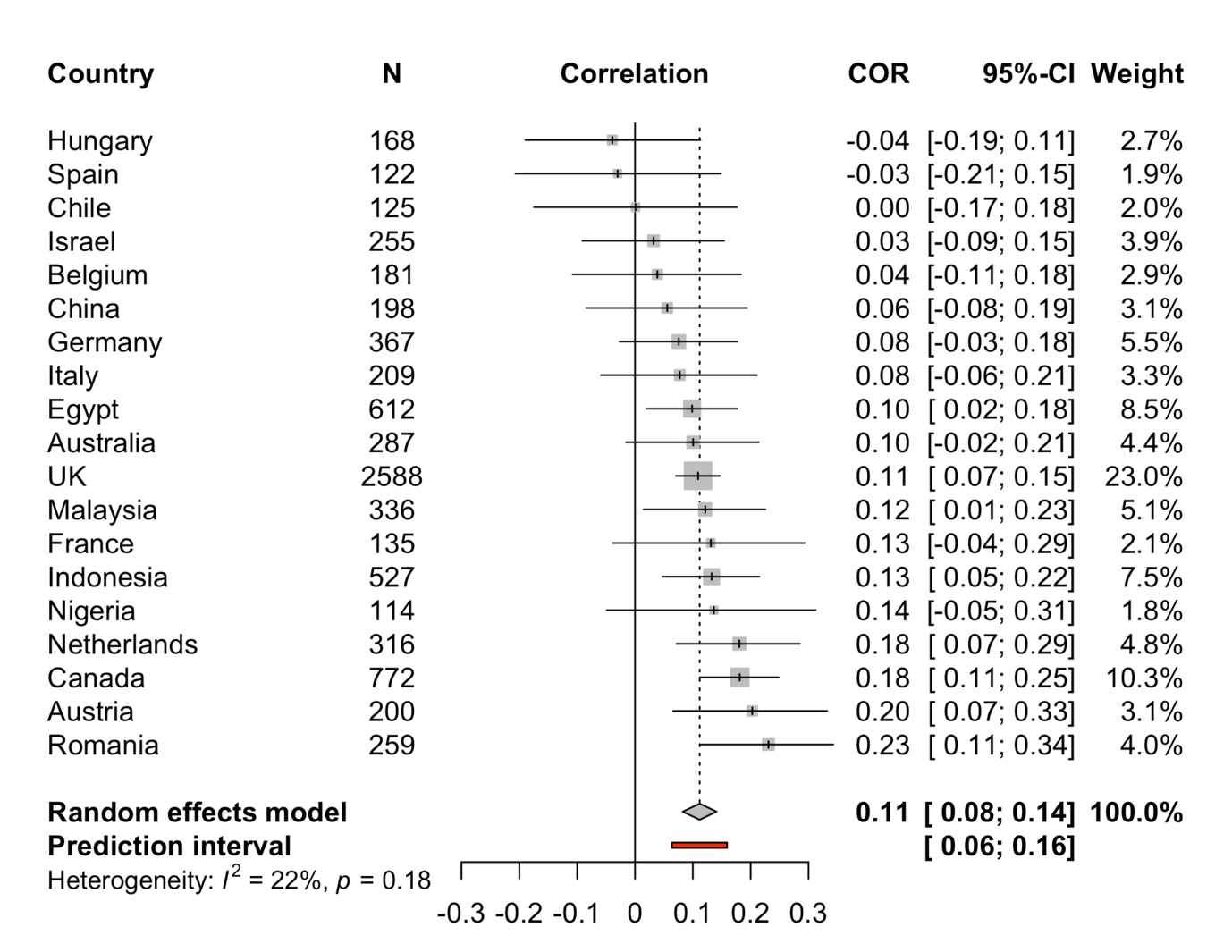


**Figure S8**: Forest plot showing random-effect meta-analysis of the relationship between intuitive CRT scores and BiG. The plot displays overall and country-specific coefficients as solid squares, with their respective 95% Confidence Intervals (CIs) displayed as error bars. The prediction interval reveals that future studies are likely to show a small positive correlation.


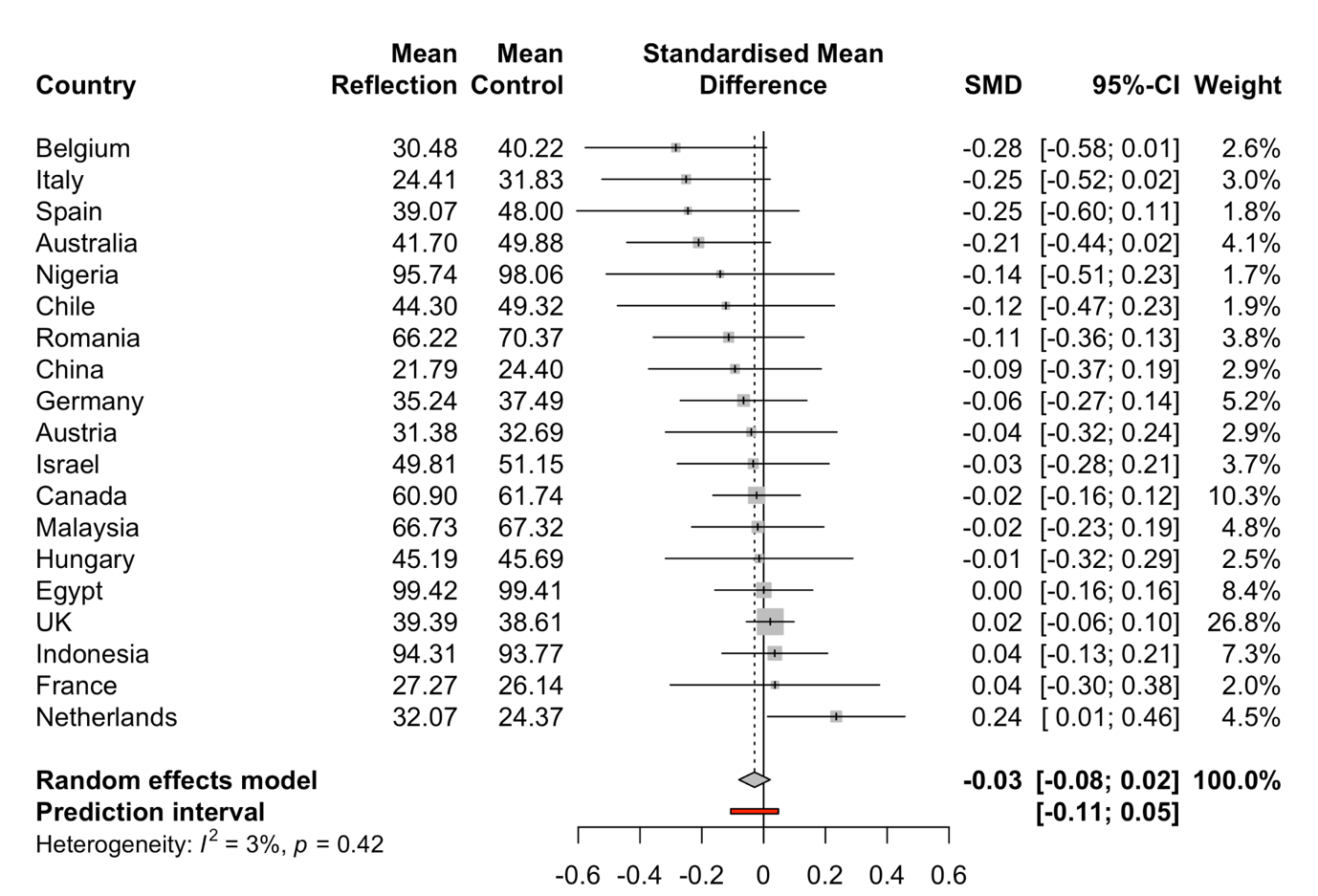


**Figure S9**: Forest plot showing random-effect meta-analysis of the standard mean differences between the reflection group (CRT presented before BiG question) and the control group (BiG questions presented before the CRT). The plot displays both overall and country-specific coefficients as solid squares, with their respective 95% Confidence Intervals (CIs) displayed as error bars. The prediction interval reveals that future studies could show a small negative or small positive correlation.


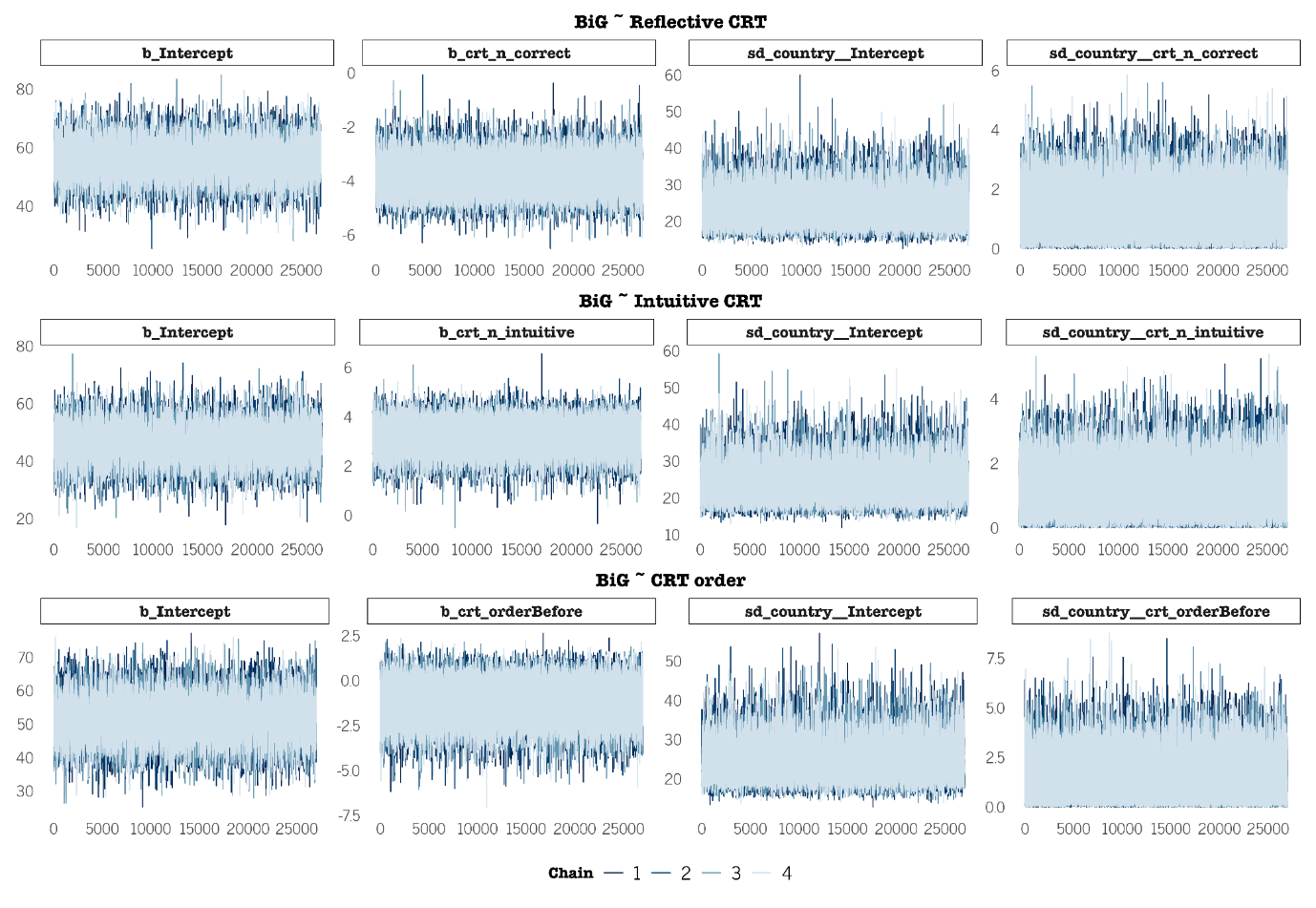
**Figure S10**: Trace plots of main effects of the Bayesian models. As shown in the figure, the trace plots confirm successful convergence of the chains. Notably, none of the population-level effects exhibited R hat values exceeding 1, providing evidence of convergence.


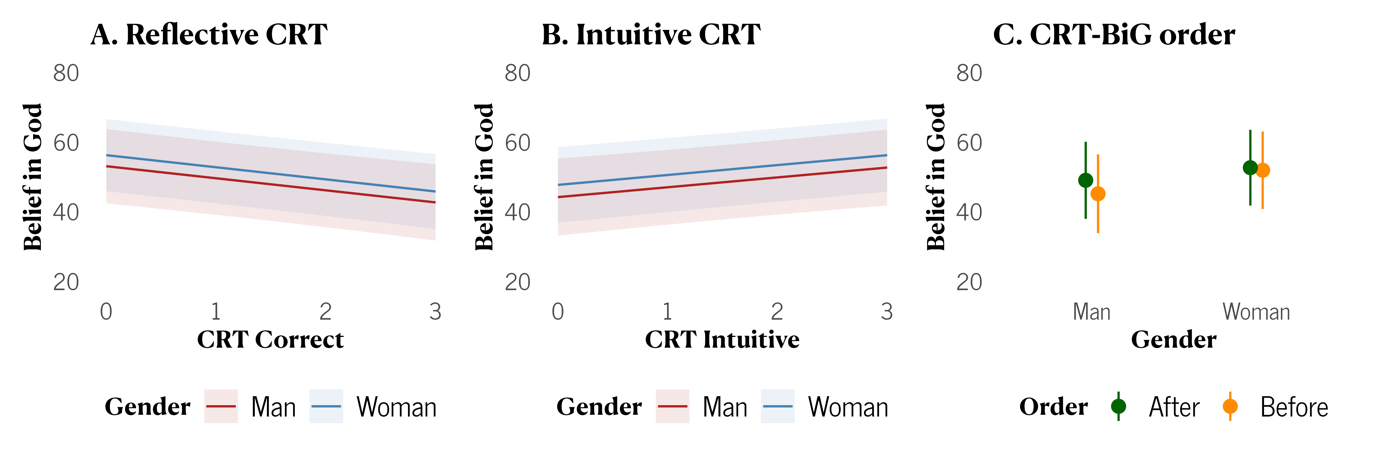


**Figure S11:** Exploratory analysis of predicted BiG as a function of CRT performance and gender, based on three frequentist hierarchical models with fixed effects for CRT performance, gender, and their interaction, and random intercepts and slopes for CRT performance by country. Panel A shows the estimated marginal effect of reflective CRT for men and women. Panel B shows the estimated marginal effect of number of intuitive CRT score for men and women. Panel C snows the estimated average effect of order (before vs. after) for men and women. Error bars represent 95% confidence intervals of the estimates.

**Table S1:** Overall and by-country number of participants (N), mean score (M), standard deviation (SD), and alpha (α) and omega (ω) reliability coefficient; and Pearson correlation (r) between the focal CRT measure and BiG. Reflective CRT scoring columns report statistics for reflective CRT scoring, and intuitive CRT columns report statistics for intuitive CRT scoring. BiG columns report means for BiG scores for all participants, participants assigned to the reflection condition, and participants assigned to the control condition.

|  |  | **Reflective CRT score (0-3)** | | | | **Intuitive CRT score (0-3)** | | | | **BiG (0-100)** | | |
| --- | --- | --- | --- | --- | --- | --- | --- | --- | --- | --- | --- | --- |
| **Country** | **N** | **M (SD)** | **α** | **ω** | **r** | **M (SD)** | **α** | **ω** | **r** | **M (SD)** | **M Reflection (SD)** | **M Control (SD** |
| Australia | 287 | 1.24 (1.1) | 0.66 | 0.68 | -0.15 | 1.56 (1.2) | 0.67 | 0.72 | 0.10 | 45.63 (38.9) | 41.70 (38.6) | 49.88 (39.0) |
| Austria | 200 | 1.75 (1.1) | 0.59 | 0.59 | -0.25 | 1.09 (1.0) | 0.57 | 0.58 | 0.20 | 32.02 (32.5) | 31.38 (33.8) | 32.69 (31.3) |
| Belgium | 181 | 1.49 (1.1) | 0.61 | 0.62 | -0.08 | 1.31 (1.1) | 0.62 | 0.66 | 0.04 | 35.06 (34.4) | 30.48 (32.4) | 40.22 (36.0) |
| Canada | 772 | 1.16 (1.1) | 0.69 | 0.70 | -0.19 | 1.64 (1.1) | 0.65 | 0.65 | 0.18 | 61.34 (37.2) | 60.90 (37.4) | 61.74 (37.0) |
| Chile | 125 | 0.82 (1.0) | 0.56 | 0.59 | -0.02 | 1.94 (1.0) | 0.53 | 0.54 | 0.00 | 46.79 (40.8) | 44.30 (39.7) | 49.32 (42.0) |
| China | 198 | 2.19 (0.9) | 0.40 | 0.49 | -0.07 | 0.43 (0.7) | 0.23 | 0.28 | 0.06 | 23.01 (28.0) | 21.79 (28.4) | 24.40 (27.6) |
| Egypt | 612 | 0.23 (0.5) | 0.45 | 0.50 | -0.05 | 2.51 (0.8) | 0.50 | 0.52 | 0.10 | 99.41 (6.6) | 99.42 (6.6) | 99.41 (6.5) |
| France | 135 | 1.27 (1.1) | 0.63 | 0.66 | -0.10 | 1.56 (1.2) | 0.70 | 0.73 | 0.13 | 26.66 (30.7) | 27.27 (29.7) | 26.14 (31.7) |
| Germany | 367 | 1.72 (1.1) | 0.62 | 0.63 | -0.10 | 1.11 (1.0) | 0.59 | 0.60 | 0.08 | 36.41 (34.6) | 35.24 (35.6) | 37.49 (33.8) |
| Hungary | 168 | 2.11 (1.1) | 0.65 | 0.68 | 0.03 | 0.74 (1.0) | 0.65 | 0.67 | -0.04 | 45.42 (34.6) | 45.19 (35.0) | 45.69 (34.3) |
| Indonesia | 527 | 0.82 (0.9) | 0.55 | 0.55 | -0.13 | 1.82 (1.0) | 0.43 | 0.45 | 0.13 | 94.04 (15.1) | 94.31 (15.2) | 93.77 (14.9) |
| Israel | 255 | 0.93 (1.0) | 0.59 | 0.60 | -0.10 | 1.82 (1.1) | 0.59 | 0.59 | 0.03 | 50.49 (39.4) | 49.81 (39.4) | 51.15 (39.6) |
| Italy | 209 | 0.93 (1.0) | 0.60 | 0.61 | -0.10 | 1.83 (1.1) | 0.62 | 0.64 | 0.08 | 28.10 (29.6) | 24.41 (27.1) | 31.83 (31.6) |
| Malaysia | 336 | 1.34 (1.1) | 0.62 | 0.63 | -0.08 | 1.47 (1.1) | 0.63 | 0.65 | 0.12 | 67.02 (30.9) | 66.73 (31.5) | 67.32 (30.4) |
| Netherlands | 316 | 1.75 (1.1) | 0.61 | 0.62 | -0.21 | 1.09 (1.0) | 0.58 | 0.60 | 0.18 | 28.56 (32.8) | 32.07 (34.3) | 24.37 (30.7) |
| Nigeria | 114 | 0.25 (0.6) | 0.79 | 0.84 | -0.36 | 2.24 (0.9) | 0.59 | 0.61 | 0.14 | 96.82 (16.4) | 95.74 (18.4) | 98.06 (13.7) |
| Romania | 259 | 1.15 (1.1) | 0.63 | 0.64 | -0.25 | 1.71 (1.1) | 0.59 | 0.63 | 0.23 | 68.31 (36.5) | 66.22 (35.9) | 70.37 (37.2) |
| Spain | 122 | 1.57 (1.2) | 0.69 | 0.69 | 0.02 | 1.20 (1.1) | 0.61 | 0.65 | -0.03 | 42.95 (36.3) | 39.07 (36.5) | 48.00 (35.8) |
| UK | 2588 | 1.12 (1.1) | 0.67 | 0.69 | -0.13 | 1.67 (1.1) | 0.65 | 0.67 | 0.11 | 39.01 (36.3) | 39.39 (36.7) | 38.61 (35.8) |
| Overall | 7771 | 1.16 (1.1) | 0.68 | 0.69 | -0.25 | 1.62 (1.1) | 0.65 | 0.67 | 0.22 | 51.96 (39.9) | 51.16 (40.0) | 52.78 (39.7) |

**Table S2**: Posterior summary of Bayesian models. The Estimate column presents the mean posterior estimate for both the overall analysis and individual countries. The HDI (Highest Density Interval) columns represent the 95% credible range of values. The rightmost column indicates the percentage of posterior draws below or above the null value of zero.

| **Country** | **Estimate** | **Lower HDI** | **Upper HDI** | **% > 0 < %** |
| --- | --- | --- | --- | --- |
| **Hypothesis 1: BiG ~ Reflective CRT** | | | | |
| **Overall** | -3.83 | -4.88 | -2.71 | 1 |
| Romania | -5.09 | -7.96 | -2.83 | 1 |
| Canada | -4.93 | -6.79 | -3.29 | 1 |
| Austria | -4.82 | -7.58 | -2.56 | 1 |
| Netherlands | -4.71 | -7.15 | -2.71 | 1 |
| Australia | -4.27 | -6.46 | -2.26 | 1 |
| Nigeria | -4.19 | -7.57 | -0.95 | 0.99 |
| UK | -4.02 | -5 | -3.03 | 1 |
| Israel | -3.86 | -6.04 | -1.52 | 1 |
| Italy | -3.7 | -6 | -1.14 | 0.99 |
| France | -3.68 | -6.08 | -0.92 | 0.99 |
| China | -3.68 | -6.11 | -0.93 | 0.99 |
| Germany | -3.62 | -5.55 | -1.49 | 1 |
| Belgium | -3.54 | -5.73 | -0.94 | 0.99 |
| Chile | -3.41 | -5.81 | -0.47 | 0.98 |
| Malaysia | -3.24 | -5.16 | -0.89 | 0.99 |
| Indonesia | -3.18 | -5.25 | -0.8 | 0.99 |
| Egypt | -3.16 | -5.78 | -0.11 | 0.97 |
| Spain | -3.03 | -5.29 | 0.12 | 0.97 |
| Hungary | -2.9 | -5.18 | 0.21 | 0.96 |
| **Hypothesis 2: BiG ~ Intuitive CRT** | | | | |
| **Overall** | 3.2 | 2.12 | 4.21 | 1 |
| Canada | 4.52 | 2.81 | 6.47 | 1 |
| Romania | 4.45 | 2.28 | 7.23 | 1 |
| Netherlands | 3.96 | 1.93 | 6.32 | 1 |
| Austria | 3.93 | 1.69 | 6.54 | 1 |
| UK | 3.42 | 2.45 | 4.4 | 1 |
| Australia | 3.29 | 1.21 | 5.27 | 1 |
| France | 3.28 | 0.83 | 5.72 | 0.99 |
| Malaysia | 3.26 | 1.23 | 5.17 | 1 |
| China | 3.2 | 0.39 | 5.8 | 0.98 |
| Nigeria | 3.17 | 0.27 | 5.95 | 0.98 |
| Germany | 3.05 | 0.9 | 5 | 0.99 |
| Italy | 2.97 | 0.47 | 5.12 | 0.99 |
| Indonesia | 2.81 | 0.61 | 4.73 | 0.99 |
| Belgium | 2.79 | 0.21 | 4.96 | 0.98 |
| Chile | 2.74 | -0.08 | 5.09 | 0.97 |
| Israel | 2.69 | 0.27 | 4.72 | 0.98 |
| Egypt | 2.57 | 0.06 | 4.72 | 0.97 |
| Spain | 2.53 | -0.42 | 4.82 | 0.95 |
| Hungary | 2.42 | -0.56 | 4.68 | 0.94 |
| **Hypothesis 3: BiG ~ CRT Order** | | | | |
| **Overall** | -1.18 | -3.01 | 0.64 | 0.09 |
| Netherlands | -0.18 | -3.42 | 3.8 | 0.38 |
| UK | -0.35 | -2.34 | 1.83 | 0.35 |
| Indonesia | -0.66 | -3.77 | 2.61 | 0.3 |
| Egypt | -0.71 | -3.85 | 2.47 | 0.29 |
| Nigeria | -0.97 | -4.91 | 2.94 | 0.26 |
| Malaysia | -0.99 | -4.14 | 2.1 | 0.22 |
| Canada | -1.01 | -3.68 | 1.6 | 0.2 |
| Hungary | -1.14 | -4.65 | 2.07 | 0.2 |
| France | -1.14 | -4.88 | 2.31 | 0.21 |
| Israel | -1.17 | -4.48 | 1.91 | 0.19 |
| Austria | -1.26 | -4.82 | 1.97 | 0.18 |
| Germany | -1.35 | -4.77 | 1.54 | 0.16 |
| Romania | -1.38 | -4.92 | 1.69 | 0.16 |
| Chile | -1.41 | -5.2 | 1.76 | 0.16 |
| China | -1.41 | -5.33 | 1.79 | 0.17 |
| Spain | -1.64 | -5.76 | 1.52 | 0.14 |
| Italy | -1.82 | -6.02 | 1.45 | 0.12 |
| Belgium | -1.91 | -6.31 | 1.24 | 0.11 |
| Australia | -1.96 | -6.09 | 1.1 | 0.09 |

**Table S3:** Summary of exploratory analyses requested by a reviewer that use frequentist hierarchical regression models. Model 1 examines the association between the number of reflective CRT responses and BIG. Model 2 explores the relationship between the number of intuitive CRT responses and BiG. Model 3 tests whether the presentation order of the CRT (before or after BiG) is associated with different BiG. All three models include random intercepts by country and random slopes for the predictor of the model. The models are equivalent to their Bayesian counterparts, employing the same fixed effects and random intercepts and slopes.

|  | **BIG ~ CRT Reflective** | | | | **BIG ~ CRT Intuitive** | | | | **BIG ~ Order** | | | | |
| --- | --- | --- | --- | --- | --- | --- | --- | --- | --- | --- | --- | --- | --- |
| *Predictors* | *Beta* | *SE* | *t* | *p* | *Beta* | *SE* | *t* | *p* | *Beta* | *SE* | *t* | *p* |  |
| (Intercept) | 55.70 | 5.24 | 10.63 | **<0.001** | 46.15 | 5.43 | 8.50 | **<0.001** | 51.55 | 5.52 | 9.34 | **<0.001** |  |
| CRT Reflective | -3.82 | 0.54 | -7.01 | **<0.001** |  |  |  |  |  |  |  |  |  |
| CRT Intuitive |  |  |  |  | 3.19 | 0.52 | 6.12 | **<0.001** |  |  |  |  |  |
| CRT Order [Before] |  |  |  |  |  |  |  |  | -1.25 | 0.87 | -1.4 | 0.185 |  |
| **Random Effects** | | | | | | | | | | | | | |
| σ^2^ | 1060.33 | | | | 1065.05 | | | | 1079.11 | | | | |
| τ_00_ | 511.20 _country_ | | | | 547.48 _country_ | | | | 571.06 _country_ | | | | |
| τ_11_ | 2.03 _country.crt_n_Reflective_ | | | | 1.75 _country.crt_n_intuitive_ | | | | 1.85 _country.crt_orderBefore_ | | | | |
| ρ_01_ | -0.03 _country_ | | | | -0.21 _country_ | | | | 0.18 _country_ | | | | |
| ICC | 0.33 | | | | 0.33 | | | | 0.35 | | | | |
| N | 19 _country_ | | | | 19 _country_ | | | | 19 _country_ | | | | |
| Observations | 7771 | | | | 7771 | | | | 7771 | | | | |
| Marginal R^2^ / Conditional R^2^ | 0.012 / 0.335 | | | | 0.008 / 0.339 | | | | 0.000 / 0.349 | | | | |
| AIC | 76295.713 | | | | 76330.005 | | | | 76426.064 | | | | |

**Table S4:** Summary of exploratory analyses requested by a reviewer that use frequentist hierarchical regression models. Model 1 examines the association between the number of reflective CRT responses, gender, their interaction, and BIG. Model 2 explores the relationship between the number of intuitive CRT responses, gender, their interaction, and BiG. Model 3 tests whether the presentation order of the CRT (before or after BiG), gender, and their interaction is associated with different BiG. All three models include random intercepts by country and random slopes for the predictor of the model. The models are equivalent to their Bayesian counterparts, employing the same fixed effects and random intercepts and slopes.

|  | **BIG ~ CRT Reflective** | | | | **BIG ~ CRT Intuitive** | | | | **BIG ~ Order** | | | |
| --- | --- | --- | --- | --- | --- | --- | --- | --- | --- | --- | --- | --- |
| *Predictors* | *Beta* | *SE* | *t* | *p* | *Beta* | *SE* | *t* | *p* | *Beta* | *SE* | *t* | *p* |
| (Intercept) | 53.08 | 5.45 | 9.74 | **<0.001** | 44.20 | 5.65 | 7.82 | **<0.001** | 49.03 | 5.65 | 8.68 | **<0.001** |
| CRT Reflective | -3.46 | 0.86 | -4.02 | **<0.001** |  |  |  |  |  |  |  |  |
| Gender [Woman] | 3.18 | 1.55 | 2.04 | **0.041** | 3.51 | 1.51 | 2.32 | **0.020** | 3.62 | 1.37 | 2.64 | **0.008** |
| CRT Cor * Gender [W] | -0.01 | 0.83 | -0.02 | 0.988 |  |  |  |  |  |  |  |  |
| CRT Intuitive |  |  |  |  | 2.83 | 0.88 | 3.22 | **0.002** |  |  |  |  |
| CRT Int * Gender [W] |  |  |  |  | 0.02 | 0.86 | 0.02 | 0.985 |  |  |  |  |
| CRT Order [Before] |  |  |  |  |  |  |  |  | -3.86 | 1.78 | -2.17 | **0.032** |
| Order [B] * Gender [W] |  |  |  |  |  |  |  |  | 3.13 | 1.92 | 1.63 | 0.104 |
| **Random Effects** | | | | | | | | | | | | |
| σ^2^ | 1058.18 | | | | 1062.44 | | | | 1073.85 | | | |
| τ_00_ | 519.64 _country_ | | | | 570.86 _country_ | | | | 575.59 _country_ | | | |
| τ_11_ | 2.11 _country.crt_n_Reflective_ | | | | 2.03 _country.crt_n_intuitive_ | | | | 2.18 _country.crt_orderBefore_ | | | |
| ρ_01_ | 0.07 _country_ | | | | -0.28 _country_ | | | | 0.36 _country_ | | | |
| ICC | 0.33 | | | | 0.34 | | | | 0.35 | | | |
| N | 19 _country_ | | | | 19 _country_ | | | | 19 _country_ | | | |
| Observations | 7647 | | | | 7647 | | | | 7647 | | | |
| Marginal R^2^ / Conditional R^2^ | 0.012 / 0.342 | | | | 0.009 / 0.346 | | | | 0.003 / 0.356 | | | |
| AIC | 75063.467 | | | | 75094.557 | | | | 75168.062 | | | |
